# From Rate-Limiting Enzyme to Therapeutic Target: The Promise of NAMPT in Neurodegenerative Diseases

**DOI:** 10.3389/fphar.2022.920113

**Published:** 2022-07-12

**Authors:** Yumeng Zhu, Ping Xu, Xuan Huang, Wen Shuai, Li Liu, Shuai Zhang, Rui Zhao, Xiuying Hu, Guan Wang

**Affiliations:** ^1^ Innovation Center of Nursing Research, West China School of Nursing, Department of Gastrointestinal Surgery, National Clinical Research Center for Geriatrics, Nursing Key Laboratory of Sichuan Province, West China Hospital, Sichuan University, Chengdu, China; ^2^ Emergency Department, Institute of Medical Big Data, Zigong Academy of Big Data for Science and Artificial Intelligence, Zigong Fourth People’s Hospital, Zigong, China

**Keywords:** nicotinamide phosphoribosyltransferase, nicotinamide adenine dinucleotide, neurodegenerative diseases, inhibitors, agonists

## Abstract

Nicotinamide phosphoribosyltransferase (NAMPT) is the rate-limiting enzyme in the nicotinamide adenine dinucleotide (NAD) salvage pathway in mammals. It is of great significance in the metabolic homeostasis and cell survival via synthesizing nicotinamide mononucleotide (NMN) through enzymatic activities, serving as a key protein involved in the host’s defense mechanism. The NAMPT metabolic pathway connects NAD-dependent sirtuin (SIRT) signaling, constituting the NAMPT–NAD–SIRT cascade, which is validated as a strong intrinsic defense system. Neurodegenerative diseases belong to the central nervous system (CNS) disease that seriously endangers human health. The World Health Organization (WHO) proposed that neurodegenerative diseases will become the second leading cause of human death in the next two decades. However, effective drugs for neurodegenerative diseases are scant. NAMPT is specifically highly expressed in the hippocampus, which mediates cell self-renewal and proliferation and oligodendrocyte synthesis by inducing the biosynthesis of NAD in neural stem cells/progenitor cells. Owing to the active biological function of NAMPT in neurogenesis, targeting NAMPT may be a powerful therapeutic strategy for neurodegenerative diseases. This study aims to review the structure and biological functions, the correlation with neurodegenerative diseases, and treatment advance of NAMPT, aiming to provide a novel idea for targeted therapy of neurodegenerative diseases.

## GRAPHICAL ABSTRACT

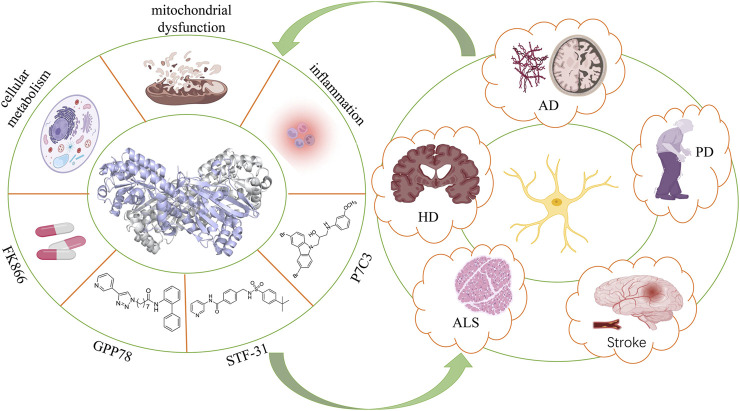

## Introduction

### Nicotinamide Phosphoribosyltransferase

The activity of NAMPT as an intracellular NAD regulator was initially reported in 1957 (Garten et al., 2015; Wang and Miao, 2015). In 1994, NAMPT was confirmed as a cytokine secreted by human peripheral blood lymphocytes (Samal et al., 1994), and it was named as the pre-B cell colony enhancing factor (PBEF) (Sun et al., 2013). Later, in 2005, NAMPT was identified to be highly expressed in visceral adipose tissues, which is closely linked with the prognosis of obese patients. Therefore, NAMPT is renamed as visfatin (Yang et al., 2006). Although NAMPT, PBEF, and visfatin have been cited in literature studies, the HUGO Gene Nomenclature Committee and the International Committee on Standardized Genetic Nomenclature for Mice both named the protein and gene of NAMPT (Fukuhara et al., 2005).

NAMPT is universally expressed in almost all organs, tissues, and cells (Yang et al., 2006), indicating the pleiotropic function in humans (Friebe et al., 2011). It is able to mediate the pathogenesis of relevant diseases by regulating inflammatory response, cell apoptosis, glucose metabolism, and oxidative stress. Therefore, NAMPT is of significance in cell metabolism, senescence, cell cycle progression, etc. Abundant homologous sequences of NAMPT exist in prokaryotes and primitive metazoans like sponges and humans (Martin et al., 2001). Both *in vitro* and *in vivo* biological activities of NAMPT have been detected (Audrito et al., 2020). *In vitro* biological functions of NAMPT as the regulator of NAD have been widely explored (Audrito et al., 2020). Through regulating the biosynthesis activity of NAD, NAMPT mediates the activities of NAD-dependent enzymes like acetylase (Garten et al., 2009; Koltai et al., 2010; Pavlova et al., 2015; Chen et al., 2016; Wang and Miao, 2019), poly(ADP-ribose) polymerase (Henning et al., 2018), and CD38 (Lee and Aarhus, 1991), thus influencing cell metabolism, mitochondrial biogenesis, and adaptive responses to inflammation and oxidation (Galli et al., 2013; Garten et al., 2015; Chen et al., 2016). Both NAMPT and its connection to the SIRT signaling constitute the powerful defense system against various stresses (Wang and Miao, 2015). SIRTs are a group of NAD-dependent histone deacetylases, the activation of which delays the occurrence of neurodegenerative diseases. Therefore, the role of SIRTs in neurological diseases has been well concerned. A previous study has proven that NAMPT delays senescence by improving cell resistance to oxidative stress (Wang et al., 2016).

NAMPT consists of extracellular and intracellular forms, called extracellular NAMPT (eNAMPT) and intracellular NAMPT (iNAMPT). eNAMPT serves as a growth factor, enzyme, and cytokine to exert its biological functions. It is an active protein in the extracellular space, first identified as PBEF and then thought to be preferentially secreted by visceral adipose tissue, called visfatin, which is produced by post-translational modifications of iNAMPT (Lundt and Ding, 2021). eNAMPT has been found in many tissues throughout the body, including the brain and blood. It is not only secreted by pre-B cells but also produced by many other cell types, such as fat cells, immune cells, brain cells, and cancer cells. Current studies suggest that eNAMPT maintains functional tissue homeostasis and enhances NAD, SIRT1 activity, and neural activation in the hypothalamus, and eNAMPT is also a major regulator of inflammatory networks, promoting the release of inflammatory cytokines (Yoshida et al., 2019; Quijada et al., 2021). Revollo’s studies found that eNAMPT and iNAMPT have similar enzymatic activities, and eNAMPT produced by differentiated adipocytes exhibits robust NAD biosynthetic activity that is even higher than iNAMPT (Revollo et al., 2007). However, based on the kinetic analyses of the enzyme, the research of Hara et al. revealed that eNAMPT does not participate in NMN formation under the extracellular milieus (Hara et al., 2011). Therefore, the mechanism and physiological function of the extracellular secretion of eNAMPT are still not particularly clear, and further research is needed in the later period (Garten et al., 2009; Travelli et al., 2018). iNAMPT is a pleiotropic protein that exists in the cytoplasm, nucleus, and mitochondria, especially in neurons of the hippocampus and cortex of the brain, and is also highly expressed in the brown adipose tissue, liver, and kidney (Garten et al., 2015). The cerebellum also expresses iNAMPT, especially in Purkinje cells, granule cells, and molecular layer cells (Liu et al., 2012). iNAMPT can be used as a key enzyme in catalyzing the biosynthetic pathway in the rate-limiting process of NAD, and it plays a variety of roles in energy metabolism, promoting endothelial cell proliferation, inhibiting apoptosis, and regulating vascular tone (Chen et al., 2015; Liu et al., 2021).

### Neurodegenerative Diseases

Neurodegenerative diseases are a type of heterogeneous disorders (Lin and Beal, 2006a), which are the biggest health issues in the global aging (Vaquer-Alicea and Diamond, 2019). They are characterized by the gradual loss of neurons that cause severe damages (Moutinho et al., 2019). Typical neurodegenerative diseases include Alzheimer’s disease (AD), Parkinson’s disease (PD), Huntington’s disease (HD), and amyotrophic lateral sclerosis (ALS) (Dugger and Dickson, 2017). At present, significant changes like inflammatory response, oxidative stress, and protein aggregation have been detected in the nerves of patients with neurodegenerative diseases (Wang et al., 2016), in which neuroinflammation and oxidative damage are prominent features of neurodegenerative diseases (Gao and Hong, 2008). So far, great efforts have been made on controlling clinical symptoms and signs of neurodegenerative diseases, but effective therapeutic strategies are lacked, which should be urgently developed and applied to clinical practice (Silvestro et al., 2021).

Normal physiological functions of neurons are highly dependent on the mitochondrial energy metabolism. Therefore, mitochondrial dysfunction is the key event of many neurodegenerative diseases (Keating, 2008). NAD is an essential coenzyme involved in energy production and redox metabolism (Lautrup et al., 2019a), which are closely linked with the mitochondrial energy metabolism. Most NADs are not synthesized *de novo*. NAMPT-mediated NAD salvage pathway is the main synthesis route of NAD, while NAD biosynthesis defects result in the decline of NAD (Imai and Guarente, 2014a). Therefore, NAMPT is vital in maintaining the homeostasis of NAD. The depletion of NAD in the brain destroys the energy homeostasis in cells and causes neuron death (Wang and Miao, 2019). Neural stem cells (NSCs) are capable of producing neurons, oligodendrocytes, or astrocytes through lineage-restricted cell division. NAMPT mediates the proliferation and aggregation of NSCs (Stein and Imai, 2014). Therefore, NAMPT is particularly important for the proliferation, self-renewal, and differentiation of neural stem/progenitor cells (NSPCs) (Wang and Miao, 2019).

In the present review, we first introduced the structure and biological functions of NAMPT, as well as the close correlation between NAMPT and neurodegenerative diseases (e.g., AD and PD). Furthermore, current agonists and inhibitors targeting NAMPT that are used for the treatment of neurodegenerative diseases were summarized for their molecule sources, characteristics, and therapeutic effects; thus, providing novel ideas for the targeted therapy of neurodegenerative diseases.

## Crystal Structure of Nicotinamide Phosphoribosyltransferase

**FIGURE 1:**
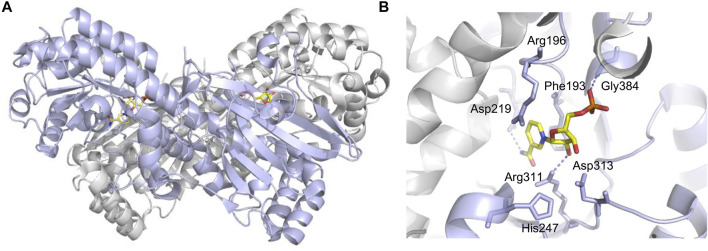
Crucial sites for NAMPT enzymatic activity (PDB code: 2H3D). **(A)** One monomer is purple, the other is gray, and NMN is shown in yellow stick shape. **(B)** Asp219 hydrogen bonded with NAM for substrate specificity. His247 is used for autophosphorylation, and dotted lines indicate hydrogen bonds.

The NAMPT gene locates on human chromosome 7q22 and spans 34.7 kb, which contains 11 exons and 10 introns, and produces a 2357-bp cDNA (Sommer et al., 2008; Sun et al., 2013). The NAMPT protein contains 491 amino acids with a molecular weight of 52 kDa (Sommer et al., 2008). Its structure is similar to that of the nicotinate phosphoribosyltransferase (NAPRTase) and quinolinate phosphoribosyl transferase (QAPRTase) of the hyperthermophilic archaea (Wang et al., 2006). NAMPT belongs to a dimeric class of type II phosphoribosyltransferases (Figure 1), the X-ray crystal structure of which confirms the low sequence identity (Wang et al., 2006). There are two active sites at the interface of the dimeric protein (Wang et al., 2006), where two NMN molecules bind. Through comparing active site sequences of 14 dimers in different organisms, including rats, the sequence of amino acid residues bound to the nicotinamide ring (substrate) and NMN (product) is identified as highly conserved (Luk et al., 2008).

## Biological Functions of Nicotinamide Phosphoribosyltransferase

In 1957, Preiss and Handler reported that NAMPT can catalyze the synthesis of nicotinamide mononucleotide (NMN) (Preiss and Handler, 1957). It is well known that NAMPT participates in the metabolism of NAD and maintains NAD levels in cells. During the past decade, the pleiotropic effect of NAMPT has been revealed. Through regulating the biosynthesis activity of NAD, NAMPT mediates the activities of NAD-dependent enzymes like poly (ADP-ribose) polymerase (PARP), ADP-ribosyltransferase (ART), and SIRT. The connection between NAMPT–NAD and SIRT constitutes a powerful defense system against various stresses (Garten et al., 2015). In addition, the production of NMN by catalyzing nicotinamide (NAM) and 5-phosphoribosyl-1-pyrophosphate (PRPP) eventually influences the activities of NAD-dependent enzymes, and as a result, NAMPT is able to mediate cell metabolism, mitogenesis, inflammation, and oxidation (Garten et al., 2015). The regulatory effect of NAMPT on SIRT has been widely concerned (Ramsey et al., 2009). Mediated by the circadian rhythm regulators CLOCK and BMAL1 of SIRT1, NAMPT can regulate the circadian rhythm of metabolism (Galli et al., 2013).

**FIGURE 2:**
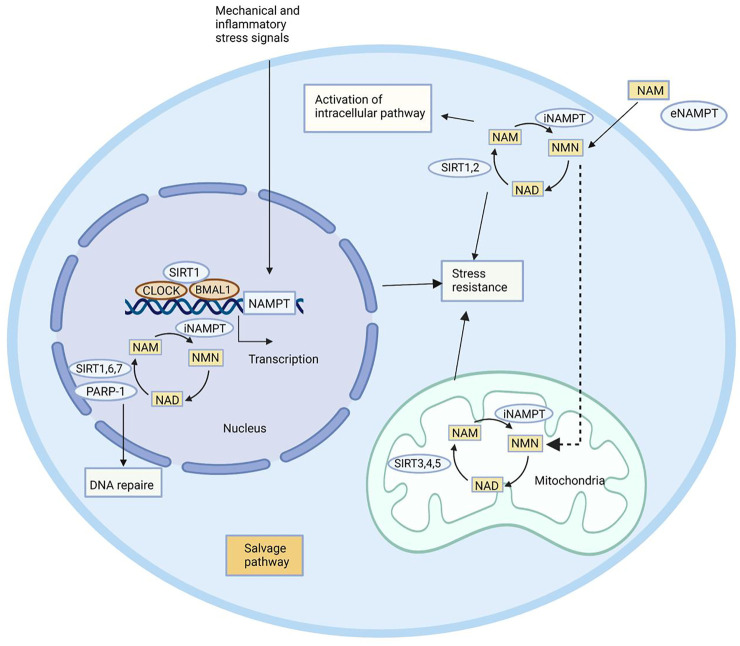
Biological functions of NAMPT. NAMPT plays its role by maintaining intracellular NAD levels and recovering NAM, which is produced by NAD-dependent enzymes PARP-1, SIRT1, SIRT6, and SIRT7 in the nucleus, SIRT1 and SIRT2 in the cytoplasm, and SIRT3, SIRT4, and SIRT5 in the mitochondria. NAMPT expression is induced by circadian regulators CLOCK and BMAL1, which are complex with SIRT1. NAMPT affects a remedial pathway that can resist stress and repair DNA. In addition to enzyme activity, NAMPT can also play an extracellular role in helping NAM produce NMN.

In addition to the intracellular functions, extracellular functions of NAMPT have been highlighted in recent years. Induced by pathogen-derived lipopolysaccharide (LPS) and host-derived inflammatory stimuli (e.g., leptin, TNF-α, IL-1β, and IL-6), NAMPT is able to mediate inflammatory response (Busso et al., 2008; Audrito et al., 2020; Wang et al., 2021). It is reported that NAMPT serves as an adipokine to regulate insulin secretion in pancreatic β-cells (Imai and Yoshino, 2013). The immune response can be influenced by NAMPT, which inhibits apoptosis of immune cells like neutrophils and macrophages (Travelli et al., 2018). Although the mechanism underlying extracellular functions of NAMPT has not been clearly demonstrated (Carbone et al., 2017), its potential as a therapeutic target has been highlighted due to vital physiological functions (Figure 2).

## The Close Correlation Between Nicotinamide Phosphoribosyltransferase and Neurodegenerative Diseases

Neurodegenerative diseases are caused by the apoptosis of neurons in the brain and spinal cord, resulting in dysfunctions with the disease aggravation (Dugger and Dickson, 2017). Damaged nerves caused by neurodegenerative diseases not only affect existing neurons but also impair the endogenous repair mechanism for cell renewal and differentiation (Wang et al., 2016). Neurodegenerative diseases are mainly associated with oxidative stress, mitochondrial dysfunction, excitotoxicity, and inflammation (Richards et al., 2018; Tofaris and Buckley, 2018). Among them, oxidative stress is considered as a vital pathogenic factor that induces the proliferation, reduction of self-renewal and differentiation, mitochondrial dysfunction, and downregulation of NAD and NAMPT levels in neurodegenerative disease cases (Lugert et al., 2010). NAD is a classic coenzyme involved in energy production (ATP) and redox metabolism, which can be generated *de novo* from tryptophan or recovered from NAM through the NAMPT-dependent salvage pathway (Rajman et al., 2018). Due to the high energy consumption and ATP synthesis rate, rapid consumption of NAD in the brain inevitably inhibits the production of ATP, destroys the energy homeostasis, and ultimately leads to neuron death (Lautrup et al., 2019a). PARPs (PAPR1 as the most analyzed), CD38 (a transmembrane enzyme), and SIRT are examined as NAD-dependent enzymes (Imai and Guarente, 2014a). The protective effect of NAD relies on SIRTs (Imai and Guarente, 2014a; Verdin, 2015). Among them, SIRT1 is the most analyzed member of SIRTs, which is responsive to the change of intracellular NAD level under various stresses owing to the deacylase activity (Wang et al., 2011). Since NAMPT is the rate-limiting enzyme of the NAD salvage pathway in mammals, it can regulate neurodegenerative diseases by maintaining NAD homeostasis, cell metabolism, and mitochondrial function.

### Nicotinamide Phosphoribosyltransferase and Alzheimer’s Disease

AD is the most common neurodegenerative disease. The prevalence of AD significantly increases with age, which mainly affects the elderly. Typical manifestations of AD include memory loss that is linked with the loss of neurons in the hippocampus and others linked with frontal lobe functions. It is generally considered that the extracellular amyloid plaques like amyloid β (Aβ) (Tagliavini et al., 1988), neurofibrillary tangles (NFT) formed by hyperphosphorylated tau protein, and loss of neurons and synapses are the main pathogenic mechanisms of AD (Lin and Beal, 2006a).

So far, only five therapeutic strategies for cognitive impairment in AD cases have been approved in the United States, including the cholinesterase inhibitors (donepezil, galantamine, and rivastigmine), NMDA receptor antagonist (memantine) (Cummings et al., 2014), and the fixed-dose combination of donepezil and memantine for moderate and severe AD (Cummings et al., 2019). However, they are only effective for alleviating clinical symptoms of AD, rather than cure or prevention. At present, multiple new drugs for AD have been analyzed, including flavonoids and rapamycin (Aso and Ferrer, 2013; Maher, 2019).

It is reported that cytokines involved in mitogenesis (e.g., NRF1, NRF2, and TFAM) are associated with neurodegenerative diseases like AD. Therefore, mitochondrial dysfunction can affect the disease condition of AD. Intervention in mitochondrial levels can improve Aβ-induced degeneration and dysfunction (Golpich et al., 2017b). Impaired mitogenesis is considered as a potential factor for AD-induced mitochondrial dysfunction (Sheng et al., 2012). Therefore, to enhance mitogenesis may be a potential pharmacological strategy for the treatment of AD. iNAMPT is important in neurodegenerative diseases through mediating mitochondria to produce energy. In addition, neuroinflammation, oxidative stress, and cholinergic neuron damage also induce neurodegeneration (Kwon and Koh, 2020a). A recent study has shown that overactivation of the immune proteasome (IP) triggers neuroinflammation and the death of neurons (Sonninen et al., 2020). Through suppressing inflammatory response and oxidative stress, iNAMPT is functionally involved in neurodegenerative diseases.

### Nicotinamide Phosphoribosyltransferase and Parkinson’s Disease

PD is the second most common neurodegenerative disease in the world, which seriously affects the normal life of middle-aged and elderly patients (Jankovic and Tan, 2020a). The necrosis of dopaminergic neurons in the substantia nigra of the midbrain is the typical pathological feature of PD. The exact pathogenic mechanism of PD, however, has not been fully elucidated (Tomas-Camardiel et al., 2004; Kalia and Lang, 2015). It is generally believed that mitochondrial dysfunction, oxidative stress, chronic inflammation, and abnormal protein folding are main causes leading to PD (Pajares et al., 2020). Latest studies have shown that increased activation of microglia in the substantia nigra of PD patients is responsible for inflammatory response (Kwon and Koh, 2020a).

Currently approved PD drugs by the U.S. Food and Drug Administration (FDA) mainly include dopaminergic antiparkinsonism agents [e.g., aromatic l-amino acid decarboxylase (AADC) inhibitors, catechol-O-methyltransferase (COMT) inhibitors, monoamine oxidase B (MAO-B) inhibitors, dopamine transporter (DAT) inhibitors, and dopamine receptor (DR) agonists] (Elsworth, 2020), 5-hydroxytryptamine (5-HT) antiparkinsonism agents [e.g., serotonin 2A (5-HT2A) receptors and serotonin 2C (5-HT2C) receptor antagonists], and cholinergic drugs [e.g., muscarinic acetylcholine receptor (mAChR) antagonists and N-methyl-D-aspartic acid (NMDA) antagonists] (Hayes, 2019; Jankovic and Tan, 2020a). However, they only significantly alleviate symptoms of PD patients in a short period, while the long-term use may result in gradually aggravated autonomic dysfunction and some ineffective or insensitive symptoms (De Virgilio et al., 2016). Therefore, it is necessary to develop new drugs with less adverse events and better therapeutic efficacy.

The significant reduction of ATP and creatine phosphate (PCr) in the core–shell polymers indicates mitochondrial dysfunction of striatal dopaminergic neurons in the early and late stages of PD (Hattingen et al., 2009). Mitochondrial dysfunction affects the respiratory chain, leading to the decreased production of ATP and increased production of free radicals (Golpich et al., 2017b). Free radicals directly inhibit the mitochondrial respiratory chain, which further leads to oxidative damage (Weise et al., 2005). iNAMPT can maintain cell metabolism, and in turn affects mitochondrial function. Studies found that mitochondrial dysfunction is a key driver of PD. Increasing NAD by nicotinamide nucleoside (NR), the precursor of NAD, significantly improved the mitochondrial function of neurons in patients. iNAMPT can utilize NAD biosynthesis enzymes NR kinase 1 (NRK1) to synthesize NAD from NAD precursors (Schondorf et al., 2018). Great efforts have been made on exploring the therapeutic potential of NSCs in PD (Zhang et al., 2017). A growing number of evidence has shown that NSCs suffer cell senescence under various stresses (Zeng et al., 2021). iNAMPT is particularly important for the proliferation, self-renewal, and differentiation of NSPCs. The NAMPT agonist P7C3-A20 is able to protect mature hippocampal neurons and substantia nigra dopaminergic neurons in the PD model (Vazquez-Rosa et al., 2020). Therefore, targeting iNAMPT would become a novel research direction for the treatment of PD.

### Nicotinamide Phosphoribosyltransferase and Ischemic Stroke

Ischemic stroke is the second leading cause of death in the world, which is the most common cause of permanent disability in adults (O'Donnell et al., 2010; Orellana-Urzua et al., 2020). Previous studies mainly analyzed the oxidative stress and inflammation in ischemic stroke cases, and relevant neuroprotective drugs based on these mechanisms have been developed (Maida et al., 2020). However, they have not been applied to clinical practice due to inappropriate selection of animal models, severe adverse events, and individualized differences (Wu et al., 2020).

Currently, ischemic stroke can only be treated with intravenous tissue plasminogen activator (t-PA) and endovascular treatment (Adeoye et al., 2011; Dirnagl and Endres, 2014). However, they are limited by the narrow range of indications, production of highly harmful reactive oxygen species (ROS), and oxidative stress, leading to brain damages (Orellana-Urzua et al., 2020). In addition, oxidative stress also causes cell apoptosis, autophagy, and necrosis in the brain (Orellana-Urzua et al., 2020). Although ischemic stroke has been extensively analyzed, the effective treatment is lacked and requires further explorations.

The interruption of cerebral arteries causes oxygen and glucose deprivation (OGD), which not only induces changes in blood vessels but also affects neuronal and glial function (Khoshnam et al., 2017). iNAMPT in the brain is mainly expressed in neurons and NSCs, which is responsible for regulating the nerve function (Wang et al., 2011; Stein and Imai, 2014). It is reported that the mean plasma eNAMPT level in ischemic stroke patients is two to eight times higher than that of non-stroke subjects (Kadoglou et al., 2014). Mitochondrial dysfunction is considered as one of the clinical signs of ischemic stroke, and therefore, targeting mitochondria may be a promising therapeutic strategy for ischemic stroke (He et al., 2020a). NAD is important in maintaining cell energy and mitogenesis. Its level decreases in the hypoxic environment after ischemic stroke due to insufficient blood supply. It is confirmed that upregulation of iNAMPT is favorable to protect from stroke (Jing et al., 2014; Wang and Miao, 2019). The connection of the NAMPT–NAD axis and SIRT forms a powerful defense system against various stresses (Wang and Miao, 2015). A growing number of *in vitro* and *in vivo* studies have demonstrated the neuroprotective effect of iNAMPT on stroke (Wang and Miao, 2015). Therefore, the role of NAMPT in cerebral ischemic injury has been well concerned, and it is expected to be applied to the treatment of ischemic stroke.

### Nicotinamide Phosphoribosyltransferase and Amyotrophic Lateral Sclerosis

ALS is a genetic neurodegenerative disease involving the upper motor neurons (brain, brain stem, and spinal cord), lower motor neurons (cranial nucleus and anterior horn cells), and the trunk, limbs, and head and face muscles they control (van Es et al., 2017). The main symptoms of ALS include muscle weakness and spasm, fasciculation and atrophy, and others include cognitive impairment, dysphonia, and salivation (Hardiman et al., 2017a). ALS affects most of the muscles including the respiratory muscles, and patients die within 3–5 years of onset due to respiratory failure (Niedermeyer et al., 2019). At present, the complicated mechanism of ALS has not been fully elucidated. Mitochondrial dysfunction, excitotoxicity, oxidative stress, metabolic disorders, and neuroinflammation are considered as potential pathological factors (Hardiman et al., 2017a; Chia et al., 2018).

ALS can be intervened by pharmacological and non-pharmacological methods. So far, more than 50 drugs with different mechanisms have been analyzed for the treatment of ALS, but only riluzole and edaravone are available in the market. Riluzole is the first drug for ALS approved by the FDA (Kiernan et al., 2011; Hardiman et al., 2017a). After 18-months treatment with riluzole, the survival of ALS patients is prolonged for 3 months, which is the only drug providing the survival benefit of about 3 months (Hobson and Mcdermott, 2016). Edaravone has been approved by the FDA as an antioxidant that alleviates the early-stage onset and disease progression of ALS. Symptomatic treatment drugs for muscle spasms like baclofen and tizanidine, both of which are muscle relaxants, are available (van Es et al., 2017). Nevertheless, most symptomatic treatments for ALS have not been tested in randomized controlled trials, and they are mainly assessed based on the management of other diseases (van Es et al., 2017). It is necessary to develop novel drugs with the high efficacy and safety for ALS.

Primary astrocytes isolated from mice overexpressing mutant human superoxide dismutase 1 (hSOD1) can induce the death of motor neurons (Harlan et al., 2016; Obrador et al., 2021). The increased mitochondrial level of NAD in astrocytes of ALS cases enhances the resistance to oxidative stress and reverses the toxicity of co-cultured motor neurons (Obrador et al., 2021). iNAMPT is the rate-limiting enzyme of the NAD salvage pathway, and its overexpression upregulates mitochondrial level of NAD in astrocytes. Therefore, iNAMPT may be a potential therapeutic target to prevent astrocyte-mediated motor neuron death in ALS cases.

### Nicotinamide Phosphoribosyltransferase and Huntington’s Disease

HD is a progressive autosomal dominant inherited neurodegenerative disease that seriously affects the quality of life (Walker, 2007). The main symptoms of HD include mental disorders, chorea, dystonia, and cognitive impairment (Walker, 2007). It is caused by an increase in trinucleotide (CAG) in the HTT gene that causes repeated polyglutamine sequence in the Huntingtin protein. Mutant Huntingtin further results in neuronal dysfunction and death via regulating cellular protein, transcription, mitochondria, and synaptic function (Harlan et al., 2019). At present, supportive and symptomatic treatment is preferred to HD patients and effective therapeutic strategies to improve the disease are lacked (Jimenez-Sanchez et al., 2017).

Mitochondrial disorders and oxidative stress are the main cellular characteristics of HD (Mccolgan and Tabrizi, 2018). Increased proliferation of cells expressing PCNA in the subventricular zone (SVZ) of HD patients after death indicates the accelerated neurogenesis (Liu and Song, 2016). SIRT1 is a NAD-dependent lysine deacetylase, and its overexpression is able to alleviate mutant Huntingtin (mHTT) toxicity and peripheral defects in the transgenic N171-82Q mice, showing a neuroprotective effect (Naia et al., 2017). The activity of SIRT1 can be regulated by iNAMPT by mediating the biosynthetic activity of NAD. Abundant evidence has proven the therapeutic potential of iNAMPT in HD. How iNAMPT regulates HD mouse models and induced pluripotent stem cells (iPSCs) derived from HD patients’ needs thorough analyses in the future.

Currently, there are no drugs that can prevent neuron death in neurodegenerative diseases. Owing to the regulatory effects on cell metabolism, mitochondrial dysfunction, inflammatory response, and oxidative stress, NAMPT has been extensively analyzed in the treatment of cancers, obesity, and other diseases (Revollo et al., 2007; Sampath et al., 2015; Zhang H. et al., 2019). However, its application in the treatment of neurodegenerative diseases has been rarely reported. So far, the significance of NAMPT in the proliferation, self-renewal, and differentiation of NSPCs has been reported (Wang et al., 2016). It is believed that NAMPT can be a promising therapeutic strategy for neurodegenerative diseases.

## Current Progress of Nicotinamide Phosphoribosyltransferase-Based Drugs

### Nicotinamide Phosphoribosyltransferase Inhibitors in Clinical Stage

In 2002, the first nanomolar inhibitor FK866 (compound **1**, also known as APO866 and WK175) appeared. Then, Hasmann reported the inhibition and antitumor effects of FK866 on NAMPT (Hasmann and Schemainda, 2003). Subsequently, the research studies showed that FK866 and nicotinamide competed for the same site, and FK866 had a higher affinity for the site (K_i_ = 0.3 nM) than niacinamide, the natural substrate of NAMPT (K_m_ = 2 µM). Early studies have shown that FK866 can significantly reduce intracellular NAD levels, resulting in delayed apoptosis of human leukemia cells and death of HEPG2 hepatoma cells (Wosikowski et al., 2002). The X-ray crystal structure of FK866 and NAMPT complex further promoted the research and development of NAMPT inhibitors (Khan et al., 2006). Currently, the NAMPT inhibitor FK866 had completed phase II trials (NCT00435084, NCT00432107, and NCT00431912). In addition, CHS828 (compound **2**, also known as GMX1778) with a similar structure had been shown to inhibit intracellular NAD synthesis (Olesen et al., 2008). Pharmacological studies demonstrated that NAMPT was the main biological target for the cytotoxic activity of GMX1778 and that GMX1778 was a competitive inhibitor (Watson et al., 2009). However, its clinical application had been limited by the problems such as short half-life, thrombocytopenia, and gastrointestinal toxicity. Finally, CHS828 was withdrawn in phase I trials (NCT00003979) (Hovstadius et al., 2002). To overcome the pharmacokinetic and solubility problems observed in early clinical trials of CHS828, a water-soluble prodrug of CHS828, EB1627, later renamed GMX1777 (compound **3**), was synthesized and it could be administered intravenously. EB1627 showed synergistic antitumor activity in mice when combined with etoposide (Binderup et al., 2005). Unfortunately, GMX1777 was withdrawn in phase I trials (NCT00457574) and terminated in phase II (NCT00724841).

**FIGURE 3:**
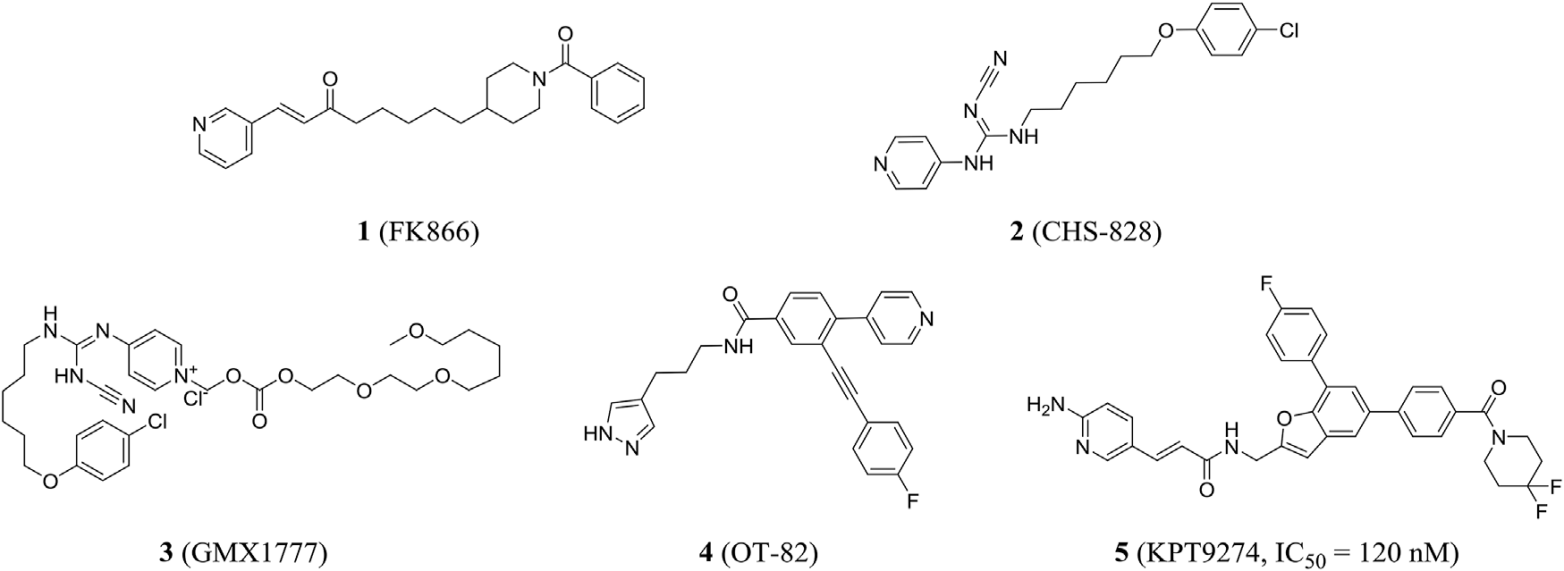
NAMPT inhibitors in clinical trials.

Recently, Korotchkina et al. identified a small molecule, OT-82 (compound **4**), characterized by pyrazole groups, that showed no cardiac, neurological, or retinal toxicity compared to other NAMPT inhibitors. Research showed strong dependence of neoplastic cells of hematopoietic origin on NAMPT; therefore, OT-82 may be used for hematological malignancies treatment. OT-82 is enrolled in a phase I clinical trial (NCT03921879) to evaluate patients for lymphoma (Korotchkina et al., 2020). KPT9274 (compound **5**) was a dual inhibitor of p21-activated kinase (PAK4) and NAMPT, and the IC_50_ of KPT9274 was about 120 nM (Figure 3). Moreover, KPT9274 showed strong cytotoxic activity against B-ALL cells. KPT-9274 was being evaluated in a phase I clinical trial in solid tumors and lymphomas (NCT02702492) (Abu et al., 2016). It is currently evaluated in phase I trials enrolling patients with advanced solid tumors or non-Hodgkin’s lymphoma acute myeloid leukemia (NCT04281420 and NCT04914845).

### Small-Molecule Inhibitors of Nicotinamide Phosphoribosyltransferase

**FIGURE 4:**
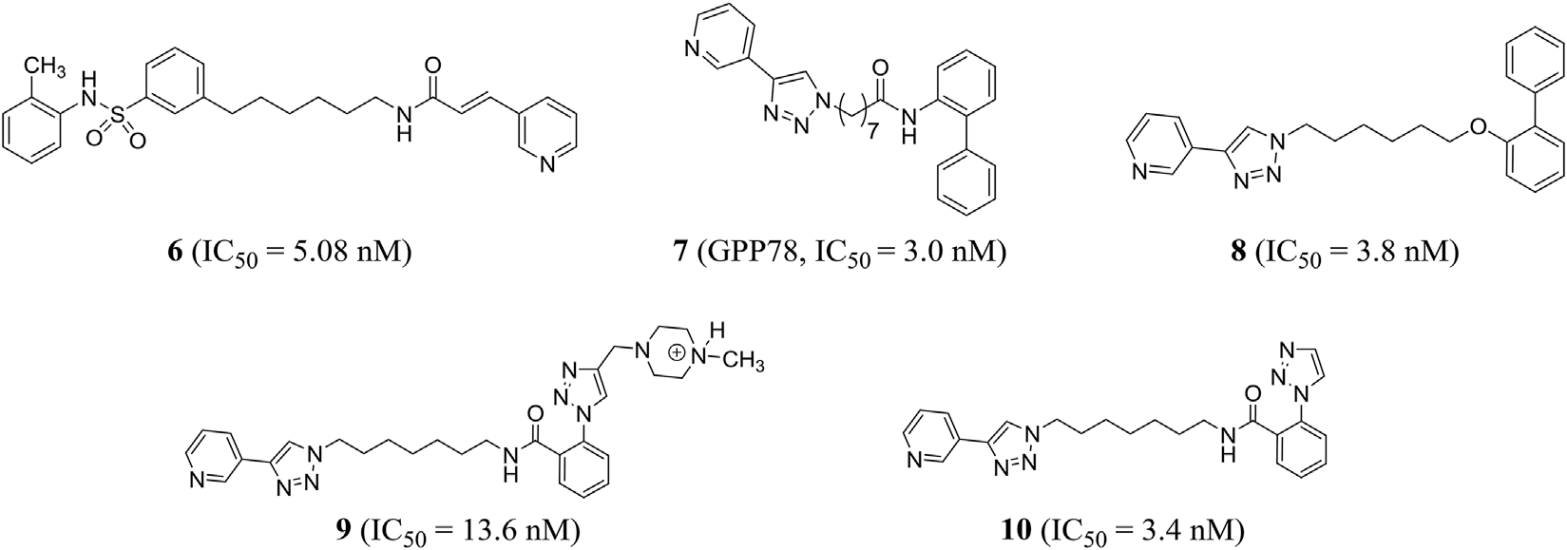
Chemical structures and kinase activities of NAMPT inhibitors **6**–**10**.

Based on the phase II NAMPT inhibitor FK866, Zhang et al. explored the structure–activity relationship and obtained the derivative compound **6** containing diaryl as a new NAMPT inhibitor (IC_50_ = 5.08 nM) (Zhang K. et al., 2019). Colombano et al. used click chemistry for compound optimization and structure–activity relationship studies. Representative compound **7** (GPP78), a triazole-based NAMPT inhibitor, reduced the IC_50_ of cellular NAD by 3.0 nM. But there were defects of low water solubility and metabolic instability (Colombano et al., 2010). On this basis, Travelli et al. reconstructed the tail group of inhibitors and obtained a novel triazoli–nicotinamide phosphate ribotransferase (NAMPT) inhibitor compound **8**, which was the most characteristic compound in the series, with an IC_50_ of 3.8 nM. It was active *in vitro* and *in vivo* (t_1/2_ = 1.89 h) and could significantly reduce the damage of colitis model (Travelli et al., 2019). With the introduction of profitable N, N-dialkyl methylamine groups, we found an experimental/pharmacological tool that specifically targets the extracellular cytokine eNAMPT inhibitor compound **9** (IC_50_ = 13.6 nM), which could not cross the plasma membrane, and can play a role in cell biology. In addition, the author stumbled upon compound **10** (IC_50_ = 3.4 nM) that crossed the plasma membrane and was cytotoxic, effectively reducing the growth of triple negative breast cancer in mice without retinal and cardiotoxicity (Travelli et al., 2017) (Figure 4).

Using high-throughput screening, Wang et al. identified a non-fluorescent compound F671-0003 (compound **11**) and a fluorescent compound M049-0244 (compound **12**), which showed excellent *in vitro* activity (IC_50_ = 85 and 170 nM, respectively) and anti-proliferation activity of HepG2 cells (IC_50_ = 1.69 and 1.95 μM, respectively). M049-0244 is an antitumor lead compound and a fluorescent probe for living cell imaging NAMPT. The pharmacokinetic characteristics and *in vivo* distribution of these inhibitors remained to be further studied (Wang et al., 2015). Based on silico screening and low-throughput screening, Takeuchi et al. found that the representative compound AS1604498 (compound **13**) was the most effective inhibitor with an IC_50_ of 44 nM, which could be used as a hit compound to develop a new treatment for NAMPT (Takeuchi et al., 2014).

**FIGURE 5:**
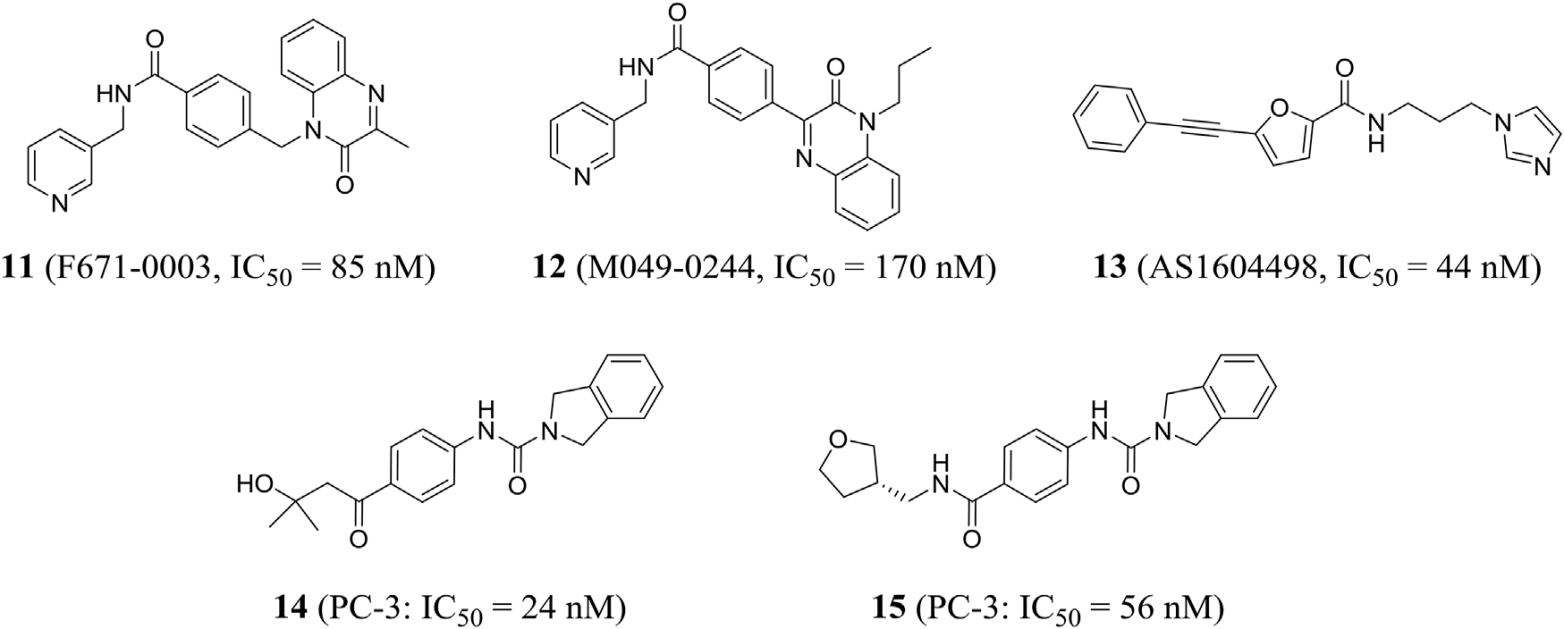
Chemical structures and activities of NAMPT inhibitors **11**–**15**.

Curtin et al. identified a variety of isoindoline analogs by using crystallographic-driven structure-based drug design and structure–activity relationship optimization. Representative compounds **14** and **15** (A-1293201) had strong anti-tumor proliferation activity, with IC_50_ values of 24 and 56 nM against PC-3 (human prostate cancer) cells, respectively (Figure 5). Both had good oral bioavailability with F values of 100 and 59%, respectively (Curtin et al., 2017). X-ray crystallography results showed that **15** isoindoline urea was partially involved in important pi-stacking interactions and hydrogen bonding and hydrophobic interactions. The authors confirmed that isoindoline urea compounds were the first known effective nonphosphate ribonucleic NAMPT inhibitors compared to FK866 (Wilsbacher et al., 2017).

Zheng et al. obtained a series of 1*H*-pyrazolo [3,4-b] pyridine-containing inhibitors through reasonable structure design. Representative compound **16** effectively inhibited NAMPT with an IC_50_ value of 6.1 nM and showed good anti-proliferation activity against A2780 cells with an IC_50_ value of 4.3 nM. In addition, compound **16** showed encouraging *in vivo* efficacy in a mouse xenograft tumor model derived from the A2780 cell line (Zheng et al., 2013a). In the same year, this group designed a class of effective amide inhibitors containing azazindole components. Representative compound **17** (GNE-617) significantly inhibited NAMPT with an IC_50_ value of 5 nM and inhibited A2780 cell proliferation with an IC_50_ value of 2 nM. The crystal structure of compound **17** and NAMPT complex revealed hydrogen bonding between amide NH and Asp219 and water-mediated hydrogen bonding between amide carbonyl group and Ser275(Zheng et al., 2013b). Subsequently, Zak et al. optimized and obtained compound **18**, which improved the time-dependent inhibition (TDI) of cytochrome P450 CYP3A4 subtype and the deficiency of low water solubility (Zak et al., 2015).

**FIGURE 6:**
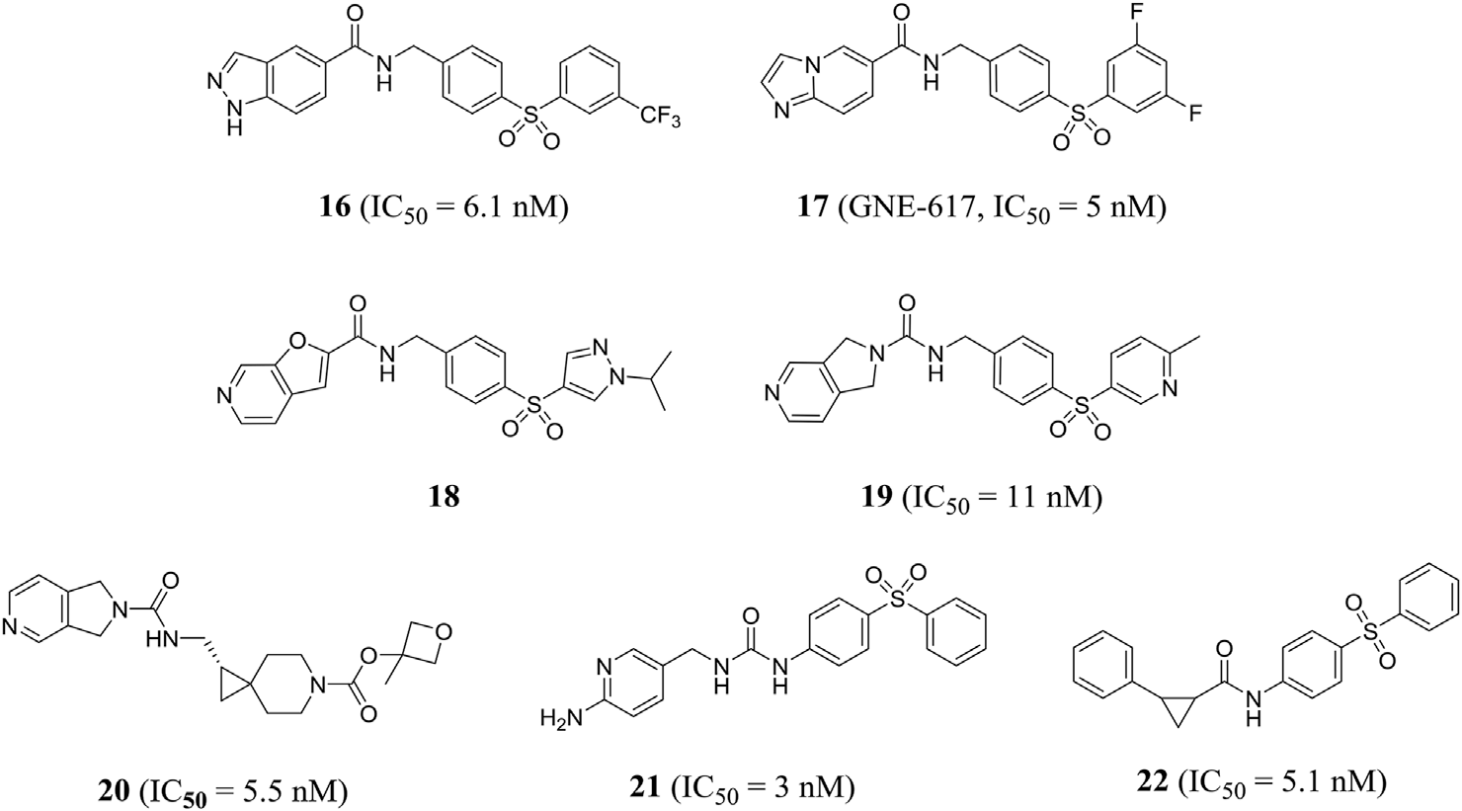
Chemical structures and kinase activities of NAMPT inhibitors **16**–**22**.

Compared with previous NAMPT inhibitors containing urea and amide, the 2, 3-dihydro-1*H*-pyrrole [3, 4-C] pyridine-derived compound **19** optimized by Dragovich et al. showed improved water solubility and strong NAMPT inhibitory activity (IC_50_ = 11 nM). PC-3 cell proliferation was effectively inhibited (IC_50_ = 36 nM) (Dragovich et al., 2013). Zak et al. found that compound 19 inhibited cytochrome P450 CYP2C9 subtype effectively (IC_50_ = 0.08 μM), which may cause unnecessary drug–drug interactions. Further optimization of pharmacodynamics and ADME properties led to the discovery of compound **20**. Compound **20** was an effective intracellular NAMPT inhibitor (IC_50_ = 5.5 nM) and had minimal inhibition on CYP2C9 (IC50 > 10 μM) (Zak et al., 2016). Gunzner-toste et al. synthesized and optimized compound **21** that did not inhibit CYP2C9, significantly inhibiting NAMPT activity (IC_50_ = 3 nM) and inhibiting A2780 cell proliferation with an IC_50_ value of 70 nM (Gunzner-Toste et al., 2013). Giannetti et al. used surface plasmon resonance (SPR) method to conduct extensive segment-based screening and structural design. Compound **22** containing *trans*-2-(pyridine-3-yl) cyclopropane-carboxamide is a representative NAMPT inhibitor (IC_50_ = 5.1 nM) (Figure 6). Encouraging *in vivo* efficacy was demonstrated in HT-1080 mouse xenograft tumor models (Giannetti et al., 2014).

Subsequently, compound **23**, which was designed by Dragovich et al. based on the fragment, exerted anti-proliferation effect on A2780 cells through NAMPT-mediated mechanism (NAMPT, IC_50_ = 0.019 μM; A2780, IC_50_ = 0.12 μM). The crystal structure showed that a hydrogen bond was observed between the amide NH portion of compound **23** and the Asp219 residue, and the water-mediated hydrogen bond was formed with two oxygen atoms in the terminal phenylsulfonamide portion (Dragovich et al., 2014). In addition, Zheng et al., through virtual screening and reasonable structural design, found that 3-pyridyl group was the preferred substitution group for inhibitor terminal, and determined that a urea moiety was the best ligand. The optimal compound **24** exhibited excellent biochemical and cellular potency (NAMPT, IC_50_ = 7 nM; A2780, IC_50_ = 32 nM) and reasonable PK properties in mice with a t_1/2_ of 1.3 h and oral bioavailability of 26% (Zheng et al., 2013c). In 2014, the author also designed a new class of cyanoguanidine-containing inhibitors containing sulfone through molecular modeling and structure–activity relationship studies. The IC_50_ value of representative compound **25** to NAMPT was 4.4 nM (Zheng et al., 2014).

**FIGURE 7:**
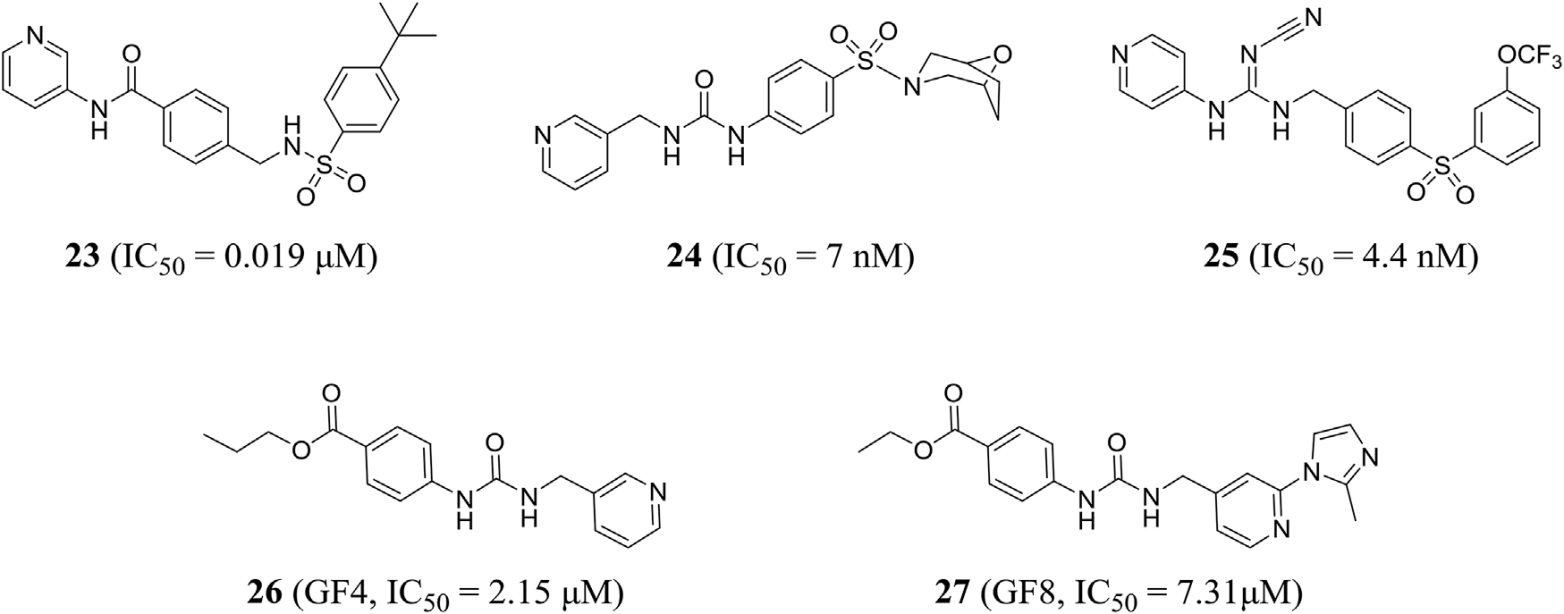
Chemical structures and kinase activities of NAMPT inhibitors **23**–**27**.

The pharmacophore-based virtual screening study by Ozgencil et al. identified compounds GF4 (compound **26**) and GF8 (compound **27**) as new urea-typed inhibitors of NAMPT with IC_50_ values of 2.15 and 7.31 μM, respectively, which also showed cytotoxicity against HepG2 cells (Figure 7). The IC_50_ values were 15.2 and 24.28 μM, respectively (Ozgencil et al., 2020).

Palacios et al. obtained compound **28** through phenotypic screening and structure-based drug design driven by crystallography, and compound **28** had good *in vivo* characteristics (t_1/2_ = 90 min). In addition, the author found that the (S, S) cyclopropylcarboxamide and the (S)-N-1-phenylethylamide were key functional groups that enhanced the activity of NAMPT inhibitors (Palacios et al., 2018). Through rational drug design, the author also found that unsubstituted pyridine provided an optimal mix of cellular potency and ADME properties, resulting in a highly potent NAMPT inhibitor **29**, which effectively inhibited A2780 cell proliferation with an IC_50_ value of 0.7 nM (Palacios et al., 2019).

**FIGURE 8:**
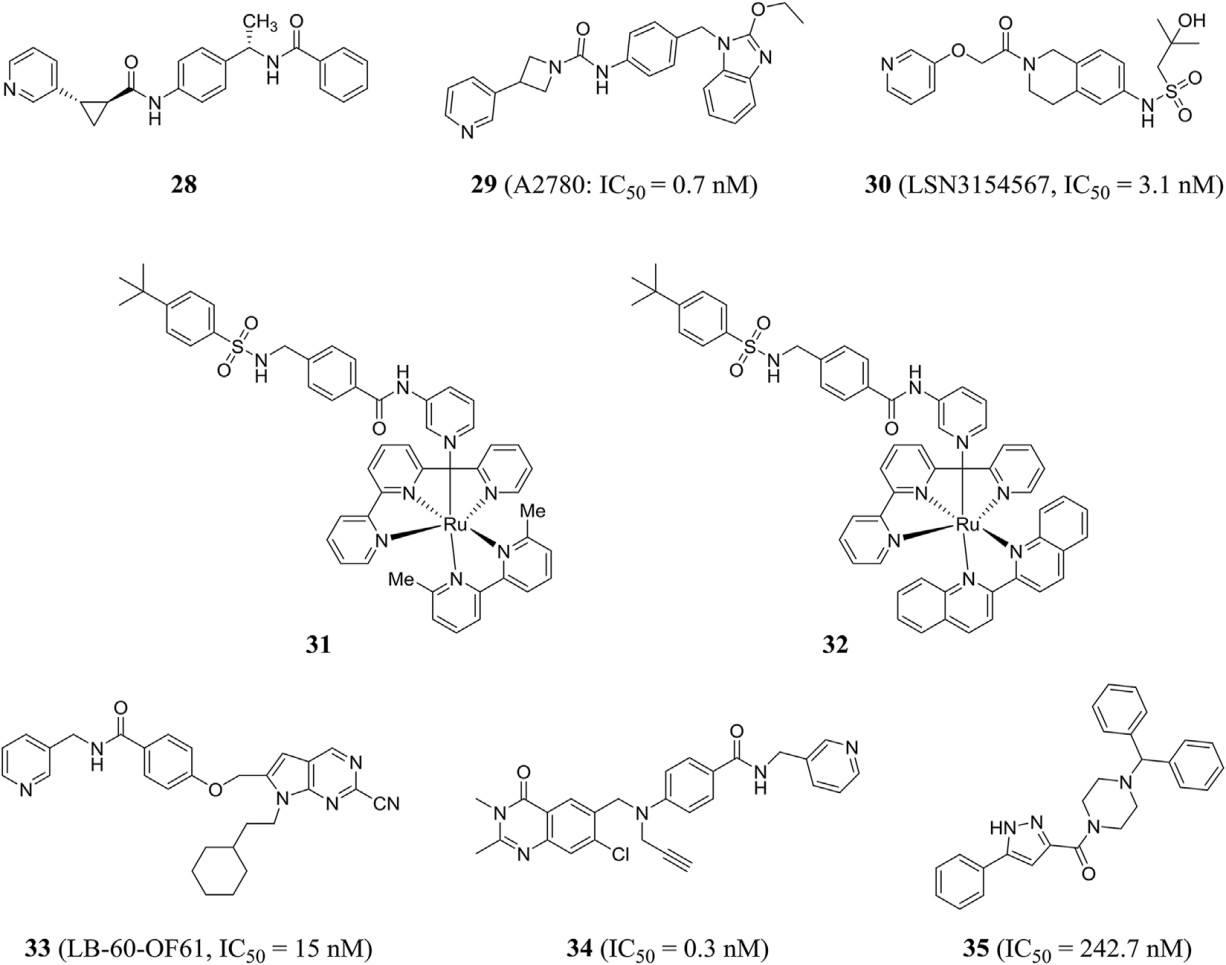
Chemical structures and kinase activities of NAMPT inhibitors **28**–**35**.

Based on virtual screening and rational structural design, a highly selective NAMPT inhibitor LSN3154567 (compound **30**) was identified, which could not cause significant retinal and hematological toxicity in rodents, but still retained strong potency (IC_50_ = 3.1 nM) (Zhao et al., 2017). Lameijer et al., using a novel prodrug strategy of photoactivated chemotherapy (PACT), obtained two water-soluble ruthenium complexes (compounds 31 and 32) that were inherently inactive. Compounds **31** and **32** had an 18-fold increase in inhibitory effect when exposed to red light (Lameijer et al., 2017). Chemical genome analysis was an effective method to elucidate the target and mechanism of compounds. Estoppey et al. pioneered the use of CRISPR/Cas9 chemogenomic profiling to identify LB-60-OF61 (compound **33**) with an IC_50_ of NAMPT about 15 nM (Estoppey et al., 2017). Lockman et al. found that 3-pyridyl formamide substituent was critical to NAMPT activity and cytotoxicity. The best compound **34** showed nanomolar NAMPT inhibition with an IC_50_ value of 0.3 nM (Lockman et al., 2010). Compound **35** was shown to be an inhibitor of NAMPT (IC_50_ = 242.7 nM) by virtual screening based on structure and ligand, and showed a protective effect when tested in axonal cell models, suggesting that inhibition of NAMPT may be an effective treatment for neurodegenerative diseases (Clark et al., 2016) (Figure 8).

### Dual-Target Inhibitors

Given the efficacy of drug combinations, and in order to overcome the pharmacokinetic problems associated with the simultaneous use of two drugs, the strategy pursued in drug development is the synthesis of hybrid molecules that exhibit two different and well-balanced mechanisms of action. With the efforts of pharmaceutical researchers, many NAMPT inhibitors with dual modes of action have made breakthroughs.

**FIGURE 9:**
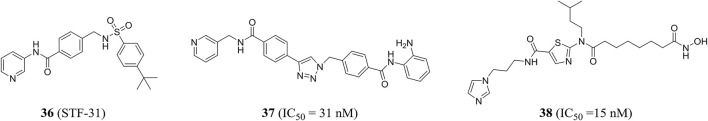
Chemical structures and kinase activities of NAMPT inhibitors **36**–**38**.

Stf-31 (compound **36**) developed by Kraus et al. has a dual mode of action and was an inhibitor of NAMPT and glucose transporter (GLUT), while the low expression level of NAMPT may be conducive to NAMPT-specific inhibition of the drug (Kraus et al., 2018). Through structure-based drug design, chemical synthesis, and biological analysis, Dong et al. successfully identified a highly efficient dual inhibitor of NAMPT and histone deacetylase (HDAC). Compound **37** showed good and balanced activities against NAMPT (IC_50_ = 31 nM) and HDAC1 (IC_50_ = 55 nM). The crystal structure showed the binding patterns of compound **37** with NAMPT and HDAC1, including π−π stacking and hydrogen bond interactions. In addition, it could effectively induce apoptosis and autophagy leading to cell death and had shown excellent *in vivo* antitumor effect in HCT116 xenograft model (Dong et al., 2017). Chen et al. in the same group also designed a class of novel NAMPT and HDAC dual inhibitors by pharmacophore fusion method, in which the representative compound **38** was highly active against the two targets (NAMPT, IC_50_ = 15 nM; HDAC1, IC_50_ = 2 nM) (Figure 9) (Chen et al., 2018).

### eNicotinamide Phosphoribosyltransferase-Neutralizing Antibody and Nicotinamide Phosphoribosyltransferasei Antibody–Drug Conjugates

Compared to traditional small-molecule NAMPT inhibitors, antibody–drug conjugates (ADCs) can selectively target cancer cells through antibodies binding to cancer-specific cell surface markers, thereby sparing normal cells from systemic NAD depletion. The dose-toxicity problems of small molecule inhibitors in clinical trials may be solved by ADCs comprising NAMPT inhibitors (NAMPTi–ADCs).

Targeting eNAMPT rather than inhibiting iNAMPT is expected to lead to safer and more effective treatments. The receptor-binding mode may be a reasonable mechanism for mediating eNAMPT cytokine-like activity. At present, toll-like receptor 4 (TLR4) is considered as putative eNAMPT receptors. eNAMPT, as a key innate immune regulator and potent damage-associated molecular pattern protein, activates an inflammatory cascade by linking TLR4 (Camp et al., 2015). Garcia’s group’s studies proved that NAMPT was strongly expressed in invasive prostate cancer tissues, especially plasma eNAMPT was significantly increased in patients with extra-prostatic invasion. eNAMPT enhanced the aggressiveness of the tumor (Sun et al., 2020). eNAMPT-neutralized humanized monoclonal antibody (ALT-100 mAb) was used to achieve effective therapeutic effect in preclinical prostate cancer (PCa) orthotopic xenotransplantation model. ALT-100 mAb not only inhibited NFκB phosphorylation and signal transduction in PCa cells *in vivo* and *in vitro* but also reduced PCa proliferation, local invasion, and distal metastasis, further inducing tumor necrosis (Sun et al., 2021). In acute respiratory distress syndrome, eNAMPT-neutralizing antibody [polyclonal antibody (pAb) or mAb] can eliminate eNAMPT-induced activation of the TLR4 pathway and disruption of the endothelial cell barrier. Intravenous administration of eNAMPT-neutralizing antibody significantly reduces the severity of acute inflammatory lung injury in mice. Therefore, eNAMPT neutralizing humanized monoclonal antibody can be used as a highly effective targeted biotherapy (Quijada et al., 2021).

eNAMPT-neutralizing antibodies are endowed with a therapeutic potential in inflammatory bowel disease. Colombo et al. reported a monoclonal antibody (C269) that neutralizes the eNAMPT cytokine-like activity while sparing the enzymatic activity. It was demonstrated that serum eNAMPT may be a predictor of anti-TNF response and that neutralization of C269 interrupts cytokines and cellular circuits that promote inflammatory bowel disease pathology in the absence of enzyme inhibition (Colombo et al., 2020). IgG-8 is a non-conjugated NAMPTi-ADC that has significantly improved toxicological characteristics. ADC delivery may provide the best form of NAMPT inhibition. ADCs have a long circulating half-life *in vivo* and may expose the drug to continuous antigen-positive target cells (Neumann et al., 2018).

### Nicotinamide Phosphoribosyltransferase Agonists in Development

Pieper and colleagues used *in vivo* screening to identify the first activator of NAMPT, P7C3 (compound **39**), with good pharmacokinetic properties and appropriate bioavailability and half-life. P7C3 protects newborn neurons from death, as well as existing mature neurons from dying in neurodegenerative diseases (Pieper et al., 2010). P7C3-A20 (compound **40**), a derivative of P7C3, also prevented neuronal cell death and increased the number of new neurons (Wang et al., 2016) and up-regulated brain NAD level during ischemic stress, playing a neuroprotective role in neurodegenerative diseases (Vazquez-Rosa et al., 2020). In 2021, Wang et al. obtained an effective NAMPT activator NAT-5r (compound **41**) through high-throughput screening and structural optimization. NAT-5r showed a K_D_ value of 132 nM and an EC_50_ value of 2.6 μM. This is the first study on the structure–activity relationship of NAMPT activator based on the co-crystal structure (Wang et al., 2022). NAT-5r can increase intracellular NAD levels and induce subsequent metabolic and transcriptional reprogramming. In addition, it effectively protects cultured cells from FK866-mediated toxicity. Strong neuroprotective efficacy in mouse models of chemotherapy-induced peripheral neuropathy without any apparent toxicity demonstrated the potential of NAMPT activator in treating neurodegenerative diseases or diseases associated with decreased NAD levels (Yao et al., 2022).

**FIGURE 10:**
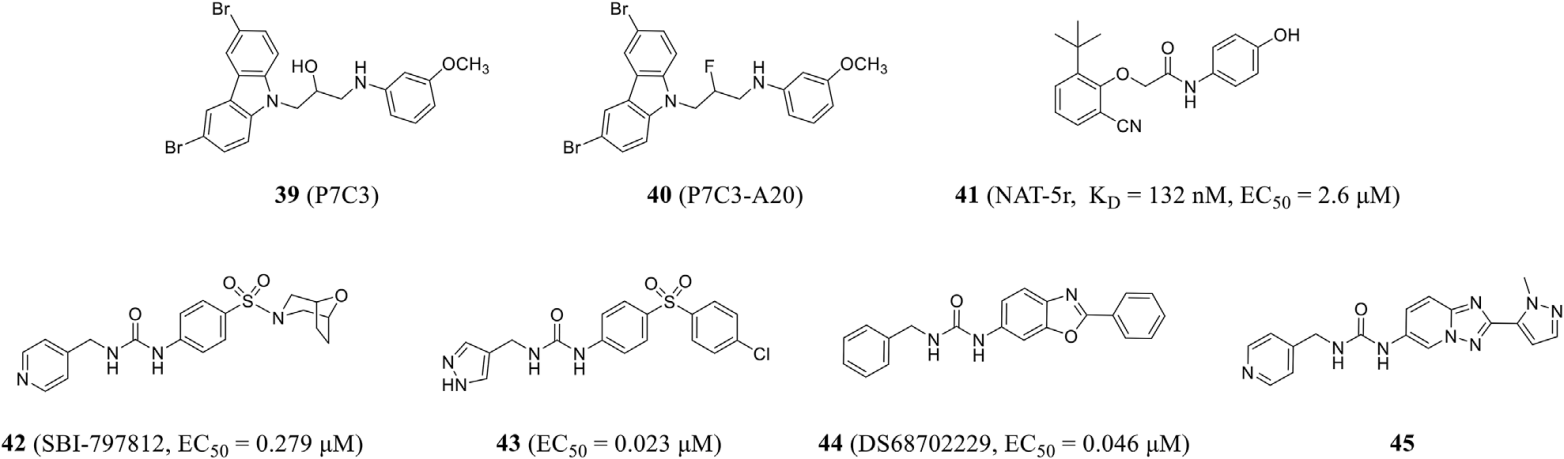
Chemical structures and kinase activities of NAMPT agonists **39**–**45**.

Gardell et al. obtained SBI-797812 (compound **42**) (EC_50_ = 0.279 μM) from a high-throughput screening of NAMPT activator and hit-to-lead campaign, which was structurally similar to active site-directed NAMPT inhibitors. The binding of these inhibitors to NAMPT could be blocked. The pyridine nitrogen was shifted from position 4 in SBI-797812 to position 2 or 3 to change the compound from NAMPT activator to inhibitor (Gardell et al., 2019). The resulting compound **43** showed more efficient NAMPT activation (EC_50_ = 0.023 μM) and improved oral bioavailability (Pinkerton et al., 2021). DS68702229 (compound **44**) was identified as a potent NAMPT activator (EC_50_ = 0.046 μM) that increases cellular NAD^+^ levels and is a promising anti-obesity drug candidate (Akiu et al., 2021a). Optimized by high-throughput screening, the same research group found pyridine-4-methyl urea derivative with double-ring core structure. SAR study proved that reducing logD was beneficial to reducing CYP inhibition, which resulted in compound **45** (Figure 10), which had the problem of low membrane permeability and still needed further optimization (Akiu et al., 2021b).

Previous studies mainly focus on the anti-cancer functions of NAMPT inhibitors, while their potential neuroprotective effect has been recently highlighted. They can improve neurodegenerative disease-induced cognitive impairment. Therefore, NAMPT inhibitors are novel candidates for the treatment of neurodegenerative diseases. At present, the research on small molecule compounds to activate NAMPT is initiated in the infant stage, which requires in-depth explorations in the future, thus benefiting people who suffer from neurodegenerative diseases.

## Summary and Prospect

Globally, millions of people are affected by neurodegenerative diseases. Current therapeutic strategies can only alleviate or control clinical symptoms and signs of neurodegenerative diseases, rather than cure them. Abundant *in vivo* and *in vitro* evidence has proven the role of NAMPT in the self-renewal and proliferation of neurons. The NAMPT–NAD–SIRT cascade, which is validated as a strong intrinsic defense system, fights against energy consumption and neuron death in neurodegenerative disease cases. Therefore, we believed that NAMPT is a very promising therapeutic target for neurodegenerative diseases.

Very latest, great efforts have been made on clarifying biological functions of NAMPT, especially the extracellular functions. Meanwhile, research on developing NAMPT agonists and inhibitors has achieved satisfactory outcomes. Small-molecule NAMPT inhibitors remarkably eliminate tumor lesions in a short period by downregulating NAD. However, effective NAMPT agonists or inhibitors in the application of neurodegenerative diseases are very scant. It is urgent and essential to clearly elucidate the mechanism underlying the neuroprotective effect of NAMPT and to develop more effective NAMPT-target drugs with less adverse events for neurodegenerative diseases.

## Glossary

5-HT: 5-Hydroxytryptamine
5-HT2A: Serotonin 2A
5-HT2C: Serotonin 2C
Aβ: Amyloid β-protein
AADC: Aromatic amino acid decarboxylase
AD: Alzheimer’s disease
ADCs: Antibody–drug conjugates
ALS: Amyotrophic lateral sclerosis
BMAL1: Aryl hydrocarbon receptor translocator-like protein 1
CAG: Canadian Association of Gastroenterology
CLOCK: Circadian locomoter output cycles protein kaput
COMT: Catechol-O-methyltransferase
DAT: Dopamine transporter
DR: Dopamine receptor
eNAMPT: Extracellular nicotinamide phosphoribosyltransferase
FDA: Food and Drug Administration
GLUT: Glucose transporter
HD: Huntington’s disease
HDAC: Histone deacetylase
hSOD 1: Human superoxide dismutase 1
IL: Interleukin
iNAMPT: Intracellular nicotinamide phosphoribosyltransferase
IP: Immune proteasome
iPSC: Induced pluripotent stem cell
LPS: Lipopolysaccharide
mAb: Monoclonal antibody
mAChR: Muscarinic acetylcholine receptor
MAO-B: Monoamine oxidase B
mHTT: Mutant Huntingtin
NAD: Nicotinamide adenine dinucleotide
NAM: Nicotinamide
NAMPT: Nicotinamide phosphoribosyltransferase
NAPRTase: Nicotinic acid phosphoribosyltransferase
NMDA: N-methyl-D-aspartic acid
NMN: Nicotinamide mononucleotide
NR: Nicotinamide nucleoside
NRF: Nuclear respiratory factor
NRK1: Nicotinamide nucleoside kinase 1
NSCs: Neural stem cells
NSPCs: Neural stem/progenitor cells
OGD: Oxygen–glucose-deprivation
pAb: Polyclonal antibody
PACT: Photo activated chemotherapy
PAK4: p21-activated kinase
PARP: Poly(ADP-ribose) polymerase
PBEF: Pre-B-cell colony-enhancing factor
PC-3: Human prostate cancer
PCa: Prostate cancer
PD: Parkinson’s disease
PRPP: 5′-Phosphoribosyl-1-pyrophosphate
QAPRTase: Quinolinic acid phosphoribosyltransferase
ROS: Reactive oxygen species
SIRT: Sirtuin
SPR: Surface plasmon resonance
SVZ: Subventricular zone
TDI: Time-dependent inhibition
TFAM: Mitochondrial transcription factor A
TLR4: Toll-like receptor 4
TNF: Tumour necrosis factor.
